# Uncertainty and bank risk in an emerging market: The moderating role of business models

**DOI:** 10.1371/journal.pone.0297973

**Published:** 2024-02-15

**Authors:** Japan Huynh, Thi Minh Hue Phan

**Affiliations:** Faculty of Finance and Banking, Ho Chi Minh City Open University, Ho Chi Minh, Vietnam; United Arab Emirates University, UNITED ARAB EMIRATES

## Abstract

The paper explores the role of business models in the link between uncertainty and bank risk. From the perspective of banks, given that future outcomes tend to be less predictable if banking uncertainty rises, we highlight a framework that a larger dispersion of bank shocks to bank-specific variables might mirror such decreased predictability as a consequence of increasing uncertainty. To compensate for the persistence of bank risk and address the endogeneity issue, we applied the system generalized method of moments (GMM) estimator as the main regressions. Analyzing a panel of commercial banks from Vietnam between 2007 and 2019, we find that higher levels of banking uncertainty may increase bank risk, as gauged by banks’ credit risk (loan loss reverses and non-performing loans) and default risk (Z-score index). This detrimental influence of uncertainty appears to be most pronounced with banks relying on pure lending, and it decreases with more non-interest income. A deeper investigation after estimating the marginal effects with plots reveals an asymmetric pattern that bank risk is immune to uncertainty in banks with the highest level of income diversification. Interestingly, we also provide evidence that uncertainty may lower the default risk level when income diversification exceeds a sufficiently high level. Our findings demonstrate that diversified business models are an efficient buffer against higher bank risk in times of increased uncertainty.

## 1. Introduction

In the past decades, a growing body of literature has positioned that uncertainty could induce significant consequences on economic cycles, and there is now increasing interest in deeply analyzing the channels through which uncertainty works. In this vein, uncertainty cannot be captured directly, so various uncertainty proxies have been proposed to measure it in an indirect manner. Accordingly, notable uncertainty measures are calculated based on stock return volatility, dispersion in forecasts, or variations in input and output prices [1]. Notably, a large body of documents prefers the keyword-counting mechanism in newspaper articles [2,3]. In general, all these measures are expected to quantify different perspectives of uncertainty. While capturing uncertainty, many recent studies have highlighted its influences on the banking system. For example, banks tend to alter lending prices [4], lose values [5], and hoard more liquidity under uncertainty [6,7]. Most notably, much research pays attention to how uncertainty drives bank lending. They present a common pattern that uncertainty harms loan growth with evidence on both macro and micro levels [8–10].

The link between economic uncertainty and bank risk is also a recently-positioned idea among academics mentioned above. Nevertheless, different from the rich evidence on the uncertainty-bank lending relationship, whether and how uncertainty drives bank risk is still limited in content and context for several key reasons. First, most of the existing studies on uncertainty and bank risk have focused entirely on aggregate uncertainty (i.e., economic policy uncertainty) or its subcomponents (e.g., financial uncertainty), but not considering other uncertainty measures from the specific perspective of financial markets, especially the banking sector [11–16]. These studies work under the assumption that such uncertainty translates into uncertainty in the banking system, which then influences the risk-taking behaviors of banks. In this regard, a new measure directly capturing the uncertainty in banking could be an excellent supplementary. Second, different risk dimensions have not been fully considered in each study. For example, a stream of literature focuses entirely on the Z-score measure to capture financial stability, while another considers only non-performing loans or other asset-based risk measures (see our review in section 2). Third, it should be noticed that for almost every study on the link between uncertainty and banking operations thus far, prior authors build up an uncertainty measure identically applied to all institutions in the same system. Thus, it is urgent to further explore banks’ varied reactions to uncertainty depending on their specific conditions. However, studies have not paid much attention to this issue. They only occasionally consider bank size, capital, and liquidity as moderating factors, which are all not enough to reflect banks’ capacity and willingness to deal with bank risk-taking [17].

In this paper, we fill these voids by examining the impacts of uncertainty on bank risk using a new uncertainty proxy dedicated to the banking system and a set of risk measures that capture credit risk and default risk. Notably, we examine how the influence of banking uncertainty on risk varies with different bank business models, characterized by the shift toward non-interest revenues and income diversification. The mediating role of business models is inspired by theoretical and empirical works. Theoretically, on the one hand, diversifying into different banking activities may bring about multiple drawbacks, such as moral hazard problems [18], exacerbated conflicts of interest [19], and “too complex to be monitored” issue [20]. On the other hand, engaging in non-traditional financial segments is beneficial due to mitigated asymmetric information problem [21] or the gain from diversification as widely accepted in the portfolio diversification theory. These reasons explain why we should expect that bank risk-taking behavior across varied business models might respond differently to changes in the uncertain environment. Empirically, the extant banking literature has mainly explored either the impacts of uncertainty on bank risk or the significance of business models in shaping bank risk [22–24]. Thus far, the work analyzing how the link between uncertainty and bank risk differ according to business models is scarce.

To conduct the work, we utilize a panel of commercial banks in Vietnam for the period 2007–2019. We measure uncertainty in the banking sector via the cross-sectional shock dispersion of key bank-level variables, following the procedure proposed by Buch et al. [25]. Our approach is motivated by the fact that future outcomes tend to be less predictable if banking uncertainty rises, hence we highlight a framework that a larger dispersion of bank shocks to bank-specific variables might mirror such decreased predictability as a consequence of increasing uncertainty [25]. We distinguish banks with different business models based on the share of non-interest income and the degree of revenue diversification across different sources.

Vietnam introduces a favorable environment to assess the above-noted tasks. It is a typical representative of emerging economies, where banks constantly hold a dominant role in the financial system and greatly contribute to economic growth [26]. Any extreme bank risk in such economies could have greater adverse effects than in countries with less bank dependence. As a stylized fact, emerging markets have a tendency to exhibit a higher level of uncertainty than developed economies [15]. Further, the consequences of uncertainty appear to be stronger in emerging markets [27]. Evidently, the Vietnamese banking sector demonstrates a discernible influence of government control, prioritizing political objectives over strictly commercial considerations. In Vietnam, banking uncertainty has surged and fluctuated significantly as a consequence of the 2008 global crisis and multiple policy reforms to restructure the banking system as well as to follow international standards in risk management. Such a scenario could amplify the repercussions of an uncertainty shock, thereby bearing noteworthy implications for the broader Vietnamese economy. Besides, after the reforms, the banking system has witnessed the heightened pressure of competition, and thus business models in the market have changed considerably [28]. Vietnamese banks have been more interested in diversifying their activities. This is also one of the critical strategies that regulators have encouraged in recent years, especially after a period of uncontrolled credit booms causing significant damages to the banking system. Although encouraged, generally the share of non-interest revenue earned by Vietnamese banks remains low, and there is more room for it to increase further in the coming time [29].

The paper displays some contributions to the literature. Fundamentally, it explores the influence of banking uncertainty on bank risk. Through the shock dispersion of bank-level factors, our uncertainty measure exhibits multiple advantages. It precisely gauges uncertainty on the operations of the banking system, which directly changes banks’ decisions and behaviors. It does not demand market data as other uncertainty indices do, nor does it suffer from the limitations of text-based uncertainty proxies regarding the quality and dependability of newspaper content [2]. Our work thus confirms and expands the understanding of the detrimental impact of uncertainty on bank risk, from the perspective of credit risk and insolvency risk. More importantly, we further test whether this impact asymmetrically varies based on different business models. In doing so, we perform our analysis with interaction terms of continuous variables (uncertainty and income measures), from which we can estimate the marginal effects of uncertainty on bank risk varying with the values of income measures after constructing plots. Our approach in this regard not only sheds new light on the critical conditioning role of business models but also considerably improves the interpretation of our estimates. Comparing our work with Bilgin et al. [30], we can argue that our approach advances theirs. The prior authors split their sample based on the degree of revenue diversification when examining its conditioning role in the effect of economic uncertainty on financial stability, thus narrowing the interpretation of results to the number of groups created and cannot consider the discrepancies across banks within the same group.

To gain these contributions, we proceed with the following sections. Section 2 discusses the theoretical influence of uncertainty, the importance of business models, and some related empirical evidence. We then explain our research framework in section 3 before we report the empirical results in section 4. Section 5 concludes the research and recommends policy implications.

## 2. Literature review

### 2.1 Uncertainty and bank risk

Uncertainty is expected to increase bank risk in several routes. Via the impact on aggregate demand, uncertainty may directly raise borrowers’ default likelihood, especially at firms with serious financial weaknesses [31,32]. This default risk of firms is likely to be translated into bank risk. The next mechanism suggests that uncertainty may lead to banks’ narrowed interest margins as a consequence of lower financing demands from firms [33] and higher funding costs of banks [34]. If banks cannot change their return target immediately, they tend to be more encouraged to prefer “high-risk, high-yield” projects to offset the lost profits [35], known as the “search for yield” incentive. The last route reveals that as it is hard for banks to precisely evaluate their future returns during periods of high uncertainty, one could argue that uncertainty may worsen the information asymmetry that banks confront. This may result in homogeneous lending behaviors [36,37]. Accordingly, such “herding behaviors” in lending strategies could cause more risks if they deviate from the banks’ core fundamentals.

In sharp contrast, an uncertain environment may also introduce competing effects. Under the “real option” concept [38], bank risk could be mitigated in uncertain times. Due to incomplete information in periods of high uncertainty, the chance of banks making improper choices tends to rise, likely encouraging banks to take on a “wait and see” plan and restrict loan supply to less creditworthy borrowers. Besides, the popular over-optimism during low uncertainty may make banks build up their asset portfolios and indebtedness excessively [39]. This may cause devastating results, thereby suggesting that bank risk should be positively linked with the uncertainty level.

Though how bank risk reacts to uncertainty shocks is theoretically ambiguous, most empirical studies consistently confirm the adverse influence on bank risk from uncertainty. Analyzing Chinese commercial banks, Chi and Li [13] find that economic policy uncertainty increases banks’ credit risks. Caglayan and Xu [11] and Karadima and Louri [40] concentrate on cross-country samples and they document that uncertainty drives non-performing loans adversely. Ng et al. [41] and Danisman et al. [14] look into US banks and then demonstrate that these banks tend to rise expected loan losses amid greater economic policy uncertainty. To refine their findings, Ng et al. [41] further indicate that the uncertainty-risk linkage is less pronounced for banks having more prudent behavior, which they capture by the ratio of bank capital; this conclusion contrasts with the work of Danisman et al. [14] who emphasizes the conditioning role of bank capital. The finding regarding the role of capital by Ng et al. [41] is confirmed in Phan et al. [12] with macro-level data.

Focusing entirely on emerging economies, Wu et al. [15] and Wu et al. [42] reveal that increased uncertainty is well translated into risk-taking of banks, also proxied by the Z-score index. Wu et al. [15] clarify that the translation is alleviated with increased holdings of liquid assets and decreased bank size. An interesting exception belongs to Wu et al. [42], who find that increased financial uncertainty is correlated with less risk, apart from the positive relationship between economic uncertainty and bank risk also witnessed simultaneously in their paper.

In summary, based on the aforementioned theoretical arguments and empirical evidence, there exists a heightened probability that uncertainty within the banking sector can amplify bank risks through various channels. To formally articulate, our initial hypothesis is expressed as follows:

Hypothesis 1. The degree of banking uncertainty exhibits a positive association with bank risk.

### 2.2 The role of business models

The literature has widely exhibited the risk implications of bank diversification. Engaging in various banking activities may generate agency costs [19,43] and accelerate moral hazard situations [18]. The “too complex to be monitored” downsides could outweigh any diversification benefit [20]. According to Deyoung and Roland [44], fee-charging activities are connected with increasing financial leverage and lower switching costs, which potentially prompt banks to make risky loans (when holding less equity capital) and perform loosened loan screening and monitoring (when establishing longer-term relationships with customers). Besides, banks with less credit exposure tend to be less conservative in credit business lines [45]. Taken together, these costs from income diversification and non-traditional activities could amplify the effects of uncertainty. In alignment with this premise, we articulate our hypothesis regarding the moderating effect of business models on the relationship between uncertainty and bank risk as follows:

Hypothesis 2A. The positive correlation between uncertainty and bank risk is more pronounced for banks characterized by greater income diversification.

Alternatively, the literature has also highlighted the benefits of income diversification. While diversifying into multiple activities, banks could obtain more information via more channels and possibly use such information for more effective loan screening and supervising [21]. Under this vein, decreased information asymmetry could weaken banks’ herding behaviors, thus compensating for the adverse impact of uncertainty. Another benefit of non-traditional segments is that if banks establish a lending relationship with their customers after earlier non-lending transactions, they could reduce the default probability of borrowers [46]. In this route, non-traditional segments are expected to alleviate the detrimental effect of uncertainty on the demand side. According to the “loss leader” proposition, increased revenue from non-traditional business areas could enhance bank lending by allowing banks to decrease interest rates [47]. This mechanism on the one hand softens the “search for yield” incentive, on the other hand it can promote the “wait and see” strategy as banks’ profit improves. According to these elaborations, we can develop a competing hypothesis for the moderating role of bank business models as follows:

Hypothesis 2B. The positive correlation between uncertainty and bank risk is less pronounced for banks characterized by greater income diversification.

We only know of the empirical research by Bilgin et al. [30] that deals with the effect of income diversification on the association between uncertainty and bank risk. The prior authors examine whether economic uncertainty drives Islamic and conventional banks’ default risks, using the World Uncertainty Index by Ahir et al. [3]. They find that uncertainty enhances conventional banks’ risk but does not alter that of Islamic counterparts. In an effort to anatomize this finding, Bilgin et al. [30] divide their samples based on both trading-based and fee-oriented institutions, and they conclude that their findings work for banks having higher income diversification. Different from their work, in our paper we evaluate the marginal effects of uncertainty on bank risk varying with the values of income diversification by building plots, thereby considerably improving the interpretation of the research results.

## 3. Empirical strategy

### 3.1 Data

The Vietnamese banking sector comprises commercial banks and policy banks. This study excludes the examination of non-profit policy banks due to their distinct operational frameworks compared to commercial banks. Data collection from commercial banks involves the exclusion of those that have been compulsorily acquired or are under exceptional control by the central bank (four banks as of the year-end of 2019). Additionally, joint-venture banks are eliminated from the study due to their small size, constituting a minor portion of the banking sector and not fully disclosing required financial data (two joint-venture banks).

As a consequence, our dataset encompasses 31 commercial banks in Vietnam spanning the period 2007–2019, subject to bank-level data availability. It is essential to highlight that, in compliance with Vietnamese banking regulations, financial reports of commercial banks have followed a standardized format subject to mandatory audits since 2007. This practice ensures the reliability of the data; moreover, only a few major banks published their financial reports before 2007 with the required data for analysis. Our bank sample covers most of the total banking assets (i.e., over 90% in any given year during the sample period), making it a good representative. We rely on the financial reports of Vietnamese commercial banks that are provided on their websites for the necessary information. Macroeconomic information is sourced from International Financial Statistics.

### 3.2 Uncertainty and business model variables

#### 3.2.1 Uncertainty measures in banking

In this study, we adopt the uncertainty proxy constructed by bank-level data and contains information referring to the banking-market-specific uncertainty. We adopt the methodology outlined by Buch et al. [25] for quantifying uncertainty, positing that heightened uncertainty corresponds to reduced predictability in future outcomes. Accordingly, a broader dispersion of shocks across key bank-level variables signifies an elevated level of uncertainty within the banking sector. It is essential to note that the computational framework proposed by Buch et al. [25] is structured to eliminate inherent bank-specific and time-varying components, enhancing the representativeness of their banking uncertainty metric in capturing the second moment of the distribution of shocks impacting bank-level variables.

Grounded in the theoretical model posited by Buch et al. [25], their investigation centers on scrutinizing the distribution of shocks influencing bank loan rates. As for the empirical model, Buch et al. [25] endorse the quantification of banking uncertainty by assessing the dispersion of shocks affecting diverse bank-level variables, encompassing banks’ asset expansion rate, short-term funding growth rate, and profit level (because this is a flow variable). These alternative variants of micro uncertainty are helpful to enhance the robustness of our findings. As suggested by Buch et al. [25], to obtain the shock dispersion, we conduct the first step to calculate shocks specific to each individual bank in each year, for our three variables separately by estimating the equation:

$$X_{i,t}=\alpha_{i}+\beta_{t}+\varepsilon_{i,t}$$ (1)

where *X*_*i*,*t*_ is the key variable at year *t* of bank *i α*_*i*_ is bank fixed effects, and *β*_*t*_ is time fixed effects. The residual of the model gives us a measure of bank-level shocks. We then perform the second step to estimate the final uncertainty proxy using the standard deviation of residuals:

$$Uncertainty_{t}=SD(\varepsilon_{i,t})$$ (2)

A higher value of this estimate reflects a greater degree of banking sector uncertainty. This proxy aims to precisely gauge uncertainty on the operations of the banking system, which will directly change banks’ decisions and behaviors.

#### 3.2.2 Business model variables

We typically distinguish banks with different business models based on the share of non-interest income and the degree of revenue diversification across different sources. For the purpose of robustness, we follow the former literature and employ three proxies to perform our task.

The first proxy is computed by net non-interest income divided by net operating income (denoted as *NII*); it is assumed to reflect any non-traditional banking business [24,48]. Net operating income includes net interest income and net non-interest income, which is the sum of income from commissions/fees, foreign exchange transactions, investment in securities, and other non-interest income sources. A specialized loan-granting bank tends to have a smaller share of non-interest income, while a bank with a non-traditional-activities-based business model is expected to have a larger share of non-interest income.

The second measure is captured via the spirit of the Herfindahl-Hirschman Index [49,50], denoted as *HHI*, calculated as follows:

$$HHI=1-[{\left(non-interest\;income\;ratio\right)}^{2}+{\left(interest\;income\;ratio\right)}^{2}]$$ (3)

And finally, we adopt the metrics of Laeven and Levine [19] to construct the third proxy, denoted as *LLI*, through the following equation:

LLI=1−|(interestincomeratio)−(non−interestincomeratio)| (4)

A larger value in our two income diversification indicators, *HHI* and *LLI*, implies a higher degree of revenue diversification, which suggests that bank business models become more diversified. To guarantee that the non-interest income ratio and income diversification metrics range from 0 to 1, observations with negative income components are eliminated [48]. From the perspective of a bank, which starts its operations with traditional activities generating interest income, an increase in income diversification could also imply a greater exposure to non-traditional activities. This justifies the use of our three alternative proxies to capture banks’ diversified business models.

#### 3.2.3 Bank risk and other variables

Similar to the literature strand on uncertainty, there is no agreement about the ideal bank risk measure thus far. Given this fact, we consider loan loss reverses and non-performing loans (as a ratio of total loans) to represent the risk profile of banks’ portfolio assets. The rationale for employing these risk proxies is that they capture credit risk that is the most essential and common form of bank risk, and they are extensively utilized as good indicators to capture bank risk in the banking literature.

In parallel with these credit risk measures, we also employ another proxy to capture banks’ default risk via the Z-score index. This index could be calculated from accounting data by the equation as follows:

$$Z-score=\frac{ROA+Capital}{\sigma (ROA)}$$ (5)

where *ROA* captures the return on asset ratio, *Capital* is the equity to asset ratio, and *σ(ROA)* is the standard deviation of the return on asset ratio. The Z-score is perceived as a reverse proxy of banks’ distance-to-default, hence a higher Z-score value suggests less default risk [51]. In line with many prior authors [15,52], we prefer a three-period rolling window to compute *σ(ROA)* and then employ the logarithm of (1 + *Z-score*) for further regressions.

Inspired by a broad strand of documents that explain bank risk, we control for multiple bank-level and macroeconomic elements as fundamental determinants of bank risk. Under the “too big to fail” view, large banks may be more reckless in their behaviors, causing potential risks to asset portfolios [53]. In another vein, the theory indicates that banks having more equity capital tends to be more prudent in investments, thus strongly encouraging them to monitor borrowers and mitigate bank risk [54]. Also, banks hoarding less liquid assets are potentially more risk-seeking [24]. So, in terms of bank-level factors, we consider bank size, capital, and liquidity. For macroeconomic conditions, we control for economic cycles and monetary policy. The motivation to include economic cycles is because favorable economic circumstances may facilitate bank operations and thus diminish bank risk [55], while the consideration of monetary policy is supported by a growing line of research referring to the bank risk-taking channel, claiming that lower interest rates may boost risk tolerance [56]. Specific constructions of all control variables are given in Table 1.

**Table 1:** Descriptive statistics.

	Obs	Min	Max	Mean	SD	Definitions
LLR	383	0.543	2.499	1.266	0.501	Loan loss reserves/Gross loans (%)
NPL	340	0.499	5.188	2.158	1.188	Non-performing loans/Gross loans (%)
Z-SCORE	355	2.625	5.892	3.947	0.872	Natural logarithm of [1+ (CAP + ROA)/sdROA], where sdROA is the standard deviation of return on assets (ROA)
NII	356	4.657	52.722	22.385	12.649	Non-interest income/Total operating income (%)
HHI	356	0.089	0.493	0.312	0.119	Income diversification degree, calculated by {1 –[(non-interest income ratio)^2^ + (interest income ratio)^2^]}
LLI	356	0.093	0.878	0.427	0.221	Income diversification degree, calculated by [1 –|(interest income ratio)–(non-interest income ratio)|]
SIZE	383	30.020	34.269	32.008	1.215	Natural logarithm of total assets
CAP	383	4.939	20.470	9.869	4.364	Capital equity/Total assets (%)
LIQ	383	5.570	36.034	17.114	9.182	Cash and dues/Total assets (%)
UNCA	383	13.427	34.091	21.936	6.747	Uncertainty from the asset shock dispersion
UNCF	383	15.995	40.931	24.226	7.890	Uncertainty from the funding shock dispersion
UNCP	383	0.674	2.058	1.273	0.386	Uncertainty from the profitability shock dispersion
GDP	383	5.247	7.130	6.245	0.640	GDP growth rate (%)
LDR	383	6.960	16.954	10.350	3.322	Short-term lending rates (%)

#### 3.2.4 Descriptive statistics

Table 1 displays the summary statistics of all variables. On average, the values of *LLR* and *NPL* are 1.266% and 2.158%, respectively, and these two credit measures display relatively high standard deviations, which are also noticed when looking at the Z-score measure. This reveals that our bank risk ratios have high volatility in the period under study. Non-interest income share has a mean value of 22.385%, confirming that Vietnamese banks’ income sources still depend on interest income segments. Likewise, small average values of income diversification measures (0.312 for *HHI* and 0.427 for *LLI*) imply that the degree of revenue diversification is not high. An examination on the statistical distribution of uncertainty measures (both ranges and standard deviations) shows a relatively large fluctuation over the years in terms of banking uncertainty levels. Other bank-level control variables possess considerable variations.

### 3.3 Econometric models

We analyze how bank risks react to uncertainty in banking depending on bank business models by regressing the specification as follows:

$$\begin{array}{l} Risk_{i,t}=\alpha_{0}+\alpha_{1}\times Risk_{i,t–1}+\alpha_{2}\times UNC_{t–1}+\alpha_{3}\times Bus_{i,t–1}\\ +\alpha_{4}\times UNC_{t–1}\times Bus_{i,t–1}+\alpha_{5}\times Bank_{i,t–1}+\alpha_{6}\times Macro_{t–1}+v_{i}+\varepsilon_{i,t} \end{array}$$ (6)

where *Risk*_*i*,*t*_ is the risk measure for bank *i* in year *t*. We include the lagged dependent variable as an independent variable to highlight the persistent nature of bank risk. *UNC* indicates banking uncertainty measures, alternatively calculated from the shock dispersion of assets (*UNCA*), funding (*UNCF*), and profits (*UNCP)*. *Bus* displays the measure of business models for each bank (*NII*, *HHI*, and *LLI*). The interaction term between two continuous variables, uncertainty and business models, captures how the marginal impact of uncertainty varies with business models. *Bank* and *Macro* contain bank-level and macroeconomic control variables, as elaborated earlier. *v*_*i*_ is the bank fixed effects, and *ε*_*i*,*t*_ is the error term. All controls are lagged by one year to reduce the potential reverse-causality problem and to better exhibit banks’ lagged responses to balance sheet and external shocks.

We apply the two-step system GMM estimator to regress our dynamic panel [57,58]. This technique can handle the endogeneity bias and yield consistent estimates, particularly in the case of omitted variables, measurement error, and reverse causality. Given our reliance on small sample size, we employ finite-sample correction procedures to ensure the reliability of estimation outcomes. Specifically, we adopt the small-sample correction of Windmeijer [59] for corrected cluster-robust standard errors, given that the resultant corrected variance estimate aligns closely with finite sample variance, thereby enhancing the precision of inferential analyses. Following the methodology of Roodman [60] in Stata, we utilize the “small” option in the “xtabond2” command to incorporate small-sample corrections to the covariance matrix estimate. Additionally, we restrict the number of instruments in the “gmm style” suboption, mitigating biases that may emerge as the number of instruments escalates toward the number of banks involved in the study.

## 4. Results

### 4.1 Baseline regressions

Before displaying the conditioning role of income models, we report the baseline results obtained from the model without the interaction terms. Tables 2–4 show the estimated results employing three bank risk measures as dependent variables. All three uncertainty measures are employed alternatively (*UNCA*, *UNCF*, and *UNCP*). In each table, the specifications include bank-level factors (columns 1–3), then expand with macroeconomic variables (columns 4–6), which is helpful to test the sensitivity of our estimates to change in control variables.

**Table 2:** The function of loan loss reserves.

	Dependent variable: Loan loss reserves					
	(1)	(2)	(3)	(4)	(5)	(6)
Lagged dependent variable	0.6452***	0.6666***	0.6598***	0.6070***	0.6213***	0.5944***
	(0.0157)	(0.0209)	(0.0241)	(0.0354)	(0.0364)	(0.0475)
UNCA	0.0113***			0.0123***		
	(0.0016)			(0.0013)		
UNCF		0.0061***			0.0087***	
		(0.0010)			(0.0010)	
UNCP			0.1782***			0.0708***
			(0.0321)			(0.0238)
NII	0.0012	0.0014*	0.0025***	0.0014*	0.0016**	0.0029***
	(0.0009)	(0.0008)	(0.0009)	(0.0008)	(0.0007)	(0.0008)
SIZE	0.0238*	0.0133	–0.0001	0.0313*	0.0199	–0.0018
	(0.0143)	(0.0137)	(0.0148)	(0.0181)	(0.0184)	(0.0192)
CAP	–0.0103***	–0.0110***	–0.0131***	–0.0139***	–0.0159***	–0.0184***
	(0.0036)	(0.0036)	(0.0037)	(0.0029)	(0.0032)	(0.0034)
LIQ	0.0010	–0.0022*	–0.0034**	–0.0014	–0.0012	–0.0008
	(0.0012)	(0.0012)	(0.0014)	(0.0010)	(0.0010)	(0.0016)
GDP				0.0573***	0.0744***	0.0488***
				(0.0112)	(0.0103)	(0.0109)
LDR				0.0223***	0.0253***	0.0279***
				(0.0046)	(0.0041)	(0.0049)
Observations	325	325	325	325	325	325
Banks	31	31	31	31	31	31
Instruments	28	28	28	30	30	30
AR(1) test	0.000	0.000	0.000	0.000	0.000	0.001
AR(2) test	0.341	0.344	0.361	0.368	0.394	0.442
Hansen test	0.320	0.324	0.343	0.235	0.246	0.245

Notes: The table presents the results of the GMM in dynamic models. Robust standard errors are in parentheses.

*, **, and *** denote statistical significance at the 10%, 5%, and 1% levels, respectively.

**Table 3:** Uncertainty and non-performing loans.

	Dependent variable: Non-performing loans					
	(1)	(2)	(3)	(4)	(5)	(6)
Lagged dependent variable	0.4493***	0.4580***	0.4708***	0.4752***	0.4930***	0.4103***
	(0.0206)	(0.0236)	(0.0206)	(0.0364)	(0.0330)	(0.0351)
UNCA	0.0360***			0.0400***		
	(0.0033)			(0.0033)		
UNCF		0.0274***			0.0367***	
		(0.0035)			(0.0029)	
UNCP			0.4119***			0.4578***
			(0.0857)			(0.0797)
NII	0.0000	0.0002	0.0020	–0.0008	–0.0004	0.0016
	(0.0019)	(0.0020)	(0.0023)	(0.0020)	(0.0020)	(0.0026)
SIZE	–0.1101**	–0.0796	–0.1275**	–0.1215**	–0.1165**	–0.1370***
	(0.0486)	(0.0533)	(0.0499)	(0.0488)	(0.0477)	(0.0523)
CAP	–0.0241**	–0.0248***	–0.0190*	–0.0267***	–0.0299***	–0.0294***
	(0.0095)	(0.0090)	(0.0108)	(0.0079)	(0.0078)	(0.0098)
LIQ	–0.0119***	–0.0051	–0.0020	–0.0194***	–0.0170***	–0.0138***
	(0.0044)	(0.0046)	(0.0048)	(0.0042)	(0.0039)	(0.0042)
GDP				0.3311***	0.3911***	0.2637***
				(0.0314)	(0.0286)	(0.0261)
LDR				0.0525***	0.0508***	0.0833***
				(0.0144)	(0.0145)	(0.0140)
Observations	280	280	280	280	280	280
Banks	31	31	31	31	31	31
Instruments	28	28	28	30	30	30
AR(1) test	0.001	0.001	0.001	0.001	0.001	0.001
AR(2) test	0.148	0.143	0.157	0.190	0.194	0.107
Hansen test	0.164	0.191	0.110	0.125	0.135	0.132

Notes: The table presents the results of the GMM in dynamic models. Robust standard errors are in parentheses.

*, **, and *** denote statistical significance at the 10%, 5%, and 1% levels, respectively.

**Table 4:** Uncertainty and default risk by Z-score index.

	Dependent variable: Z-SCORE					
	(1)	(2)	(3)	(4)	(5)	(6)
Lagged dependent variable	0.5790***	0.5858***	0.6229***	0.4636***	0.4945***	0.4848***
	(0.0341)	(0.0271)	(0.0226)	(0.0413)	(0.0407)	(0.0426)
UNCA	–0.0116***			–0.0063**		
	(0.0033)			(0.0032)		
UNCF		–0.0133***			–0.0104**	
		(0.0030)			(0.0042)	
UNCP			–0.0104			–0.0685
			(0.0644)			(0.0769)
NII	–0.0113***	–0.0118***	–0.0123***	–0.0031	–0.0050	–0.0086***
	(0.0012)	(0.0016)	(0.0017)	(0.0030)	(0.0032)	(0.0029)
SIZE	0.0610***	0.0482***	0.0687***	0.0479**	0.0395**	0.0438**
	(0.0147)	(0.0173)	(0.0172)	(0.0198)	(0.0184)	(0.0194)
LIQ	0.0010	0.0020	–0.0018	0.0055	0.0056	–0.0010
	(0.0029)	(0.0033)	(0.0034)	(0.0039)	(0.0058)	(0.0046)
GDP				0.1094***	0.0994**	0.1821***
				(0.0415)	(0.0465)	(0.0522)
LDR				–0.0345***	–0.0264**	–0.0318***
				(0.0092)	(0.0111)	(0.0102)
Observations	298	298	298	298	298	298
Banks	31	31	31	31	31	31
Instruments	27	27	27	29	29	29
AR(1) test	0.000	0.000	0.000	0.000	0.000	0.000
AR(2) test	0.110	0.127	0.194	0.142	0.155	0.157
Hansen test	0.304	0.264	0.301	0.191	0.206	0.351

Notes: The table presents the results of the GMM in dynamic models. Robust standard errors are in parentheses.

** and *** denote statistical significance at the 5% and 1% levels, respectively. We drop the *Capital* variable since its inclusion in the specification model of *Z-SCORE* may cause spurious regressions (*Capital* is a component of *Z-SCORE* computation). See Table 1 for the definitions of each variable.

Across all regressions in Tables 2 and 3, we show that the coefficients on uncertainty measures are positive and statistically significant, implying that banks’ credit risk is more likely to increase in times of more uncertainty. The regressions of alternative uncertainty measures remain unchanged after controlling for different sets of regressors. Quantitatively, the magnitude of the impact is also meaningful. Taking an example from the uncertainty measure using the dispersion of asset shocks, one standard deviation rise in *UNCA* (6.747) leads to a rise of 0.083 percentage points (0.0123*6.747) of loan loss reserve ratio (column 4 of Table 2) or a rise of 0.270 percentage points (0.0400*6.747) of non-performing loan ratio (column 4 of Table 3).

We turn to the discussion for the regression results for default risk. Due to the use of the three-year rolling window, our observations in the model with Z-score index slightly decrease. We document that uncertainty variables induce a significantly negative correlation with the dependent variable. Since a larger Z-score indicates a lower level of default risk, the negative coefficient estimates are described by the conclusion that banking uncertainty is found to increase banks’ default risk. This conclusion is observed when using the dispersion of shocks to assets and funding, while the coefficient of the dispersion of profit shocks is still negative but insignificant. Economically, our findings are also significant. For example, a one standard deviation rise in *UNCA* (6.747) and *UNCF* (7.890) may cause an increase in banks’ default risk of 0.043% (0.0063*6.747) and 0.082% (0.0104*7.890), respectively (columns 4 and 5 of Table 4).

Overall, we find that bank risk, in terms of credit risk and default risk, considerably increases in periods of increased uncertainty in banking. This finding verifies Hypothesis 1 and confirms the prior works that display the detrimental consequences of different uncertainty aspects, such as economic policy uncertainty [11–14] and economic uncertainty [15]. We also expand this literature strand by a new uncertainty measure that captures banking sector uncertainty.

### 4.2 Marginal effects depending on bank business models

We first explore business models’ role in the link between uncertainty and credit risk. Table 5 reports the results with *NII*, Table 6 displays the estimates with *HHI*, and Table 7 exhibits the regressions with *LLI*. Across all columns in three tables, the coefficients of standalone uncertainty measures are consistently positive and significant, thus strongly confirming the influence of banking sector uncertainty on credit risk.

**Table 5:** The interaction of banking uncertainty and non-interest income share.

	Dependent variable: Loan loss reserves			Dependent variable: Non-performing loans		
	(1) UNCA	(2) UNCF	(3) UNCP	(4) UNCA	(5) UNCF	(6) UNCP
Lagged dependent variable	0.6100***	0.5920***	0.6165***	0.4793***	0.5228***	0.4108***
	(0.0290)	(0.0490)	(0.0237)	(0.0266)	(0.0175)	(0.0357)
Uncertainty	0.0219***	0.0093***	0.1487***	0.0147***	0.0399***	0.4993***
	(0.0018)	(0.0011)	(0.0326)	(0.0048)	(0.0034)	(0.0924)
Uncertainty*NII	–0.0002***	–0.0002***	–0.0026**	–0.0007***	–0.0008***	–0.0084**
	(0.0001)	(0.0001)	(0.0011)	(0.0003)	(0.0002)	(0.0042)
NII	0.0067***	0.0047***	0.0037***	0.0049**	0.0055***	0.0079**
	(0.0016)	(0.0009)	(0.0008)	(0.0023)	(0.0014)	(0.0040)
SIZE	–0.0062	–0.0119	0.0047	–0.0160	–0.1527**	–0.1453***
	(0.0158)	(0.0145)	(0.0151)	(0.0750)	(0.0689)	(0.0519)
CAP	–0.0167***	–0.0185***	–0.0159***	0.0286	–0.0362**	–0.0308***
	(0.0030)	(0.0025)	(0.0033)	(0.0186)	(0.0152)	(0.0100)
LIQ	–0.0130***	–0.0034***	–0.0041***	–0.0010	–0.0152***	–0.0151***
	(0.0027)	(0.0012)	(0.0011)	(0.0048)	(0.0048)	(0.0042)
GDP	0.0876***	0.0841***	–0.0161	0.2785***	0.4209***	0.2563***
	(0.0135)	(0.0079)	(0.0109)	(0.0439)	(0.0432)	(0.0308)
LDR	0.0276***	0.0245***	0.0421***	0.0643***	0.0789***	0.0872***
	(0.0038)	(0.0048)	(0.0026)	(0.0163)	(0.0143)	(0.0131)
Observations	325	325	325	280	280	280
Banks	31	31	31	31	31	31
Instruments	31	31	31	31	31	31
AR(1) test	0.001	0.000	0.000	0.001	0.001	0.001
AR(2) test	0.494	0.311	0.433	0.198	0.133	0.108
Hansen test	0.306	0.279	0.402	0.149	0.227	0.142

Notes: The table presents the results of the GMM in dynamic models. The uncertainty measure employed in each regression is shown at the top of each column (UNCA, UNCF, and UNCP). Robust standard errors are in parentheses.

** and *** denote statistical significance at the 5% and 1% levels, respectively.

**Table 6:** The interaction of banking uncertainty and HHI income diversification.

	Dependent variable: Loan loss reserves				Dependent variable: Non-performing loans	
	(1) UNCA	(2) UNCF	(3) UNCP	(4) UNCA	(5) UNCF	(6) UNCP
Lagged dependent variable	0.6138***	0.5988***	0.5742***	0.5051***	0.5313***	0.4048***
	(0.0460)	(0.0419)	(0.0488)	(0.0217)	(0.0173)	(0.0372)
Uncertainty	0.0175***	0.0102***	0.1399***	0.0296***	0.0422***	0.5060***
	(0.0023)	(0.0010)	(0.0450)	(0.0064)	(0.0034)	(0.0764)
Uncertainty*HHI	–0.0168***	–0.0175***	–0.4111***	–0.0904***	–0.0796***	–0.8042***
	(0.0063)	(0.0049)	(0.1118)	(0.0217)	(0.0118)	(0.2140)
HHI	0.3119***	0.4106***	0.4369***	0.5155**	0.5953***	0.5935*
	(0.0883)	(0.0970)	(0.0889)	(0.2034)	(0.2231)	(0.3344)
SIZE	0.0106	–0.0140	0.0172	0.0179	–0.1080**	–0.1415***
	(0.0133)	(0.0158)	(0.0180)	(0.0756)	(0.0504)	(0.0530)
CAP	–0.0153***	–0.0186***	–0.0148***	0.0332*	–0.0310**	–0.0303***
	(0.0026)	(0.0027)	(0.0029)	(0.0171)	(0.0130)	(0.0098)
LIQ	–0.0060**	–0.0025**	–0.0002	0.0047	–0.0134***	–0.0158***
	(0.0026)	(0.0011)	(0.0010)	(0.0046)	(0.0046)	(0.0038)
GDP	0.0859***	0.0885***	0.0518***	0.2760***	0.4248***	0.2553***
	(0.0125)	(0.0091)	(0.0099)	(0.0456)	(0.0375)	(0.0304)
LDR	0.0207***	0.0248***	0.0276***	0.0543***	0.0926***	0.0905***
	(0.0058)	(0.0045)	(0.0050)	(0.0150)	(0.0119)	(0.0131)
Observations	325	325	325	280	280	280
Banks	31	31	31	31	31	31
Instruments	31	31	31	31	31	31
AR(1) test	0.000	0.000	0.000	0.001	0.002	0.001
AR(2) test	0.539	0.331	0.287	0.119	0.167	0.117
Hansen test	0.265	0.273	0.281	0.161	0.217	0.152

Notes: The table presents the results of the GMM in dynamic models. The uncertainty measure employed in each regression is shown at the top of each column (UNCA, UNCF, and UNCP). Robust standard errors are in parentheses.

*, **, and *** denote statistical significance at the 10%, 5%, and 1% levels, respectively.

**Table 7:** The interaction of banking uncertainty and LLI income diversification.

	Dependent variable: Loan loss reserves			Dependent variable: Non-performing loans		
	(1) UNCA	(2) UNCF	(3) UNCP	(4) UNCA	(5) UNCF	(6) UNCP
Lagged dependent variable	0.6226***	0.6270***	0.5910***	0.4967***	0.5292***	0.4033***
	(0.0436)	(0.0400)	(0.0433)	(0.0235)	(0.0176)	(0.0372)
Uncertainty	0.0154***	0.0097***	0.1621***	0.0210***	0.0390***	0.5077***
	(0.0019)	(0.0011)	(0.0342)	(0.0056)	(0.0036)	(0.0769)
Uncertainty*LLI	–0.0096***	–0.0083***	–0.3199***	–0.0479***	–0.0385***	–0.6345***
	(0.0030)	(0.0026)	(0.0585)	(0.0125)	(0.0081)	(0.1427)
LLI	0.2290***	0.2707***	0.3350***	0.3139***	0.3112***	0.5363***
	(0.0522)	(0.0649)	(0.0581)	(0.1110)	(0.0725)	(0.1930)
SIZE	0.0059	–0.0229*	0.0065	0.0064	–0.1532**	–0.1393***
	(0.0125)	(0.0137)	(0.0169)	(0.0749)	(0.0687)	(0.0531)
CAP	–0.0159***	–0.0204***	–0.0163***	–0.0326*	–0.0382**	–0.0299***
	(0.0025)	(0.0026)	(0.0032)	(0.0176)	(0.0151)	(0.0099)
LIQ	–0.0066**	–0.0031**	–0.0006	0.0026	–0.0146***	–0.0160***
	(0.0027)	(0.0012)	(0.0012)	(0.0046)	(0.0048)	(0.0039)
GDP	0.0876***	0.0872***	0.0567***	0.2903***	0.4199***	0.2508***
	(0.0121)	(0.0082)	(0.0098)	(0.0455)	(0.0400)	(0.0304)
LDR	0.0224***	0.0216***	0.0266***	0.0622***	0.0788***	0.0928***
	(0.0057)	(0.0036)	(0.0046)	(0.0160)	(0.0124)	(0.0139)
Observations	325	325	325	280	280	280
Banks	31	31	31	31	31	31
Instruments	31	31	31	31	31	31
AR(1) test	0.000	0.000	0.000	0.001	0.001	0.001
AR(2) test	0.455	0.282	0.227	0.102	0.130	0.111
Hansen test	0.267	0.254	0.303	0.158	0.230	0.155

Notes: The table presents the results of the GMM in dynamic panel models. The uncertainty measure employed in each regression is shown at the top of each column (UNCA, UNCF, and UNCP). Robust standard errors are in parentheses.

*, **, and *** denote statistical significance at the 10%, 5%, and 1% levels, respectively.

In Table 5, we find that the coefficients on the interaction term are significantly negative, possibly suggesting that the uncertainty-risk linkage is less prominent in banks with a higher non-interest revenue ratio. Turning to Tables 6 and 7, the coefficients of the interaction terms between income diversification and uncertainty are statistically significant and negative. This result may determine that the detrimental effect of uncertainty on credit risk may be alleviated by increasing the level of revenue diversification.

However, because all three income measures are continuous variables and take infinite values, the marginal effects in Eq (6) should change with the values of income measures. Hence, we have to take the derivative of Eq (6) with respect to uncertainty and construct plots to describe our estimates adequately.

**Fig 1:**
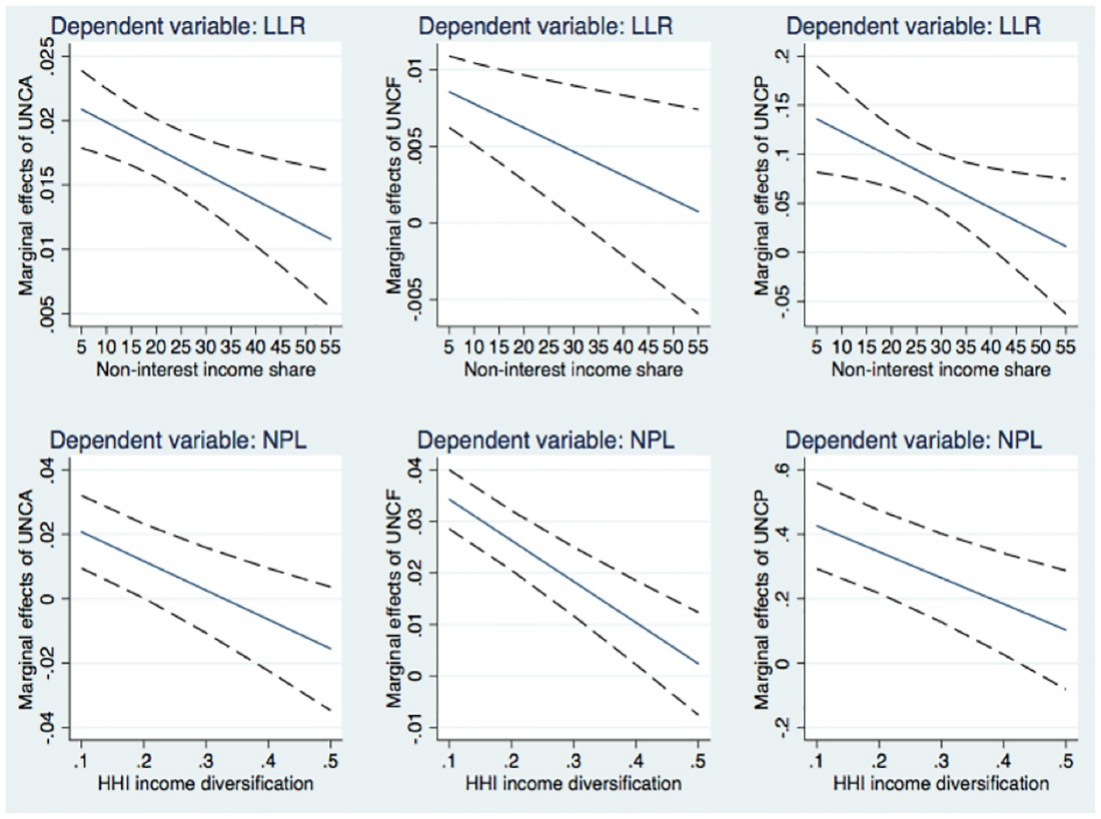
The marginal effects of banking uncertainty on credit risk in relation to business models. *Notes*: *The marginal effects on LLR are calculated with the estimates reported in columns 1–3 of* Table 5, *and those on NPL are based on the results presented in columns 4–6 of* Table 6.

Fig 1 depicts the marginal effects of uncertainty on bank risk according to the values of non-interest income ratio (*NII*) and revenue diversity (*HHI*). The dash lines illustrate the 95% confidence interval. For the use of *NII*, we find that an increase in the non-interest income share may reduce the marginal effects of uncertainty on bank risk. This pattern holds for all banks when using *UNCA* as an uncertainty measure, for banks whose *NII* value is below 31.25% when using *UNCF*, and for banks whose *NII* value is below 40.75% when using *UNCP*. For a non-interest income ratio of 31.25%/40.75% or higher, the marginal impacts are insignificant as for the 95% confidence interval, the upper limit is more than zero while the lower limit is less than zero. These banks cover only 20% of the sample banks, prompting us to confidently conclude that more reliance on non-interest income reduces the negative impact of uncertainty on bank risk. The trends in the graphs based on the *HHI* measure are similar. When the level of income diversification is not too high, our findings suggest that income diversification could likewise mitigate the detrimental influence of uncertainty. When the degree of revenue diversification is high (greater than 0.205–0.425 depending on the uncertainty measures used), a change in income diversification plays no significant role in shaping the way uncertainty drives bank risk. An illustration with the *LLI* income diversification still yields the same pattern, but we do not report it for the sake of brevity. It is always available upon request.

Our results with the unusual approach of plotting the marginal effects could offer more insight into how uncertainty asymmetrically drives bank risk. Notably, our approach advances the work of Bilgin et al. [30], who simply split the sample based on the level of income diversification before concluding about its conditioning role. Multiple potential mechanisms could explain our result. One key benefit of diversified business models is through reducing risk due to product diversification, close in the spirit with the logic of portfolio diversification theory. This upside reduces pressure on the credit segment that carries the most risk of banks. In other words, considering the transmission effectiveness of uncertainty via bank credit aspects in general (i.e., the quantity and quality of bank lending), a business model less dependent on lending activities is expected to weaken the transmission. Besides, in the context of higher uncertainty, banks are required to apply more stringent screening and monitoring styles on borrowers and loans to prevent the deterioration of asset quality. This process could be facilitated by the benefit of decreased information asymmetry when banks diversify their activities [21]. In some cases, when these benefits from diversified business models are sufficient to protect banks against the harm of uncertainty, bank risk appears not to be driven by higher uncertainty.

We now discuss the estimated results on the conditioning role of income models in the link between uncertainty and banks’ default risk. As shown in all columns in Table 8, the coefficient on the standalone uncertainty measures is significantly negative, thus firmly confirming that banks’ default risk increases in reaction to higher uncertainty in banking. For the interaction terms, their coefficients are positive and highly significant, regardless of the income measures and uncertainty variables used. These results at least suggest that business models play an essential role in moderating the impact of uncertainty on bank risk. To deeply interpret our findings, we also assess the marginal impacts of uncertainty on banks’ default risk according to banks’ business models.

**Table 8:** Default risk, banking uncertainty, and income structure.

	Dependent variable: Z-SCORE								
	Business model: NII			Business model: HHI			Business model: LLI		
	(1) UNCA	(2) UNCF	(3) UNCP	(4) UNCA	(5) UNCF	(6) UNCP	(7) UNCA	(8) UNCF	(9) UNCP
Lagged dependent variable	0.5505***	0.4699***	0.4881***	0.5485***	0.4586***	0.5076***	0.5462***	0.4643***	0.5029***
	(0.0322)	(0.0320)	(0.0362)	(0.0328)	(0.0341)	(0.0373)	(0.0300)	(0.0312)	(0.0382)
Uncertainty	–0.0404***	–0.0249***	–0.2790***	–0.0838***	–0.0403***	–0.2908***	–0.0547***	–0.0335***	–0.2884***
	(0.0141)	(0.0083)	(0.0656)	(0.0135)	(0.0142)	(0.0668)	(0.0108)	(0.0116)	(0.0607)
Uncertainty*Business model	0.0018***	0.0011***	0.0130***	0.2702***	0.1256***	1.2587***	0.1252***	0.0744***	0.8701***
	(0.0006)	(0.0004)	(0.0030)	(0.0437)	(0.0428)	(0.2962)	(0.0249)	(0.0244)	(0.1874)
Business model	–0.0557***	–0.0377***	–0.0205***	–7.8049***	–4.0185***	–1.8592***	–3.7401***	–2.3530***	–1.1583***
	(0.0111)	(0.0092)	(0.0030)	(0.6610)	(1.0327)	(0.3042)	(0.4713)	(0.5706)	(0.1822)
SIZE	0.0524***	0.0504**	0.0562***	0.0805***	0.0560***	0.0564***	0.0623***	0.0521**	0.0540***
	(0.0164)	(0.0202)	(0.0190)	(0.0102)	(0.0217)	(0.0191)	(0.0129)	(0.0210)	(0.0183)
LIQ	–0.0079	0.0005	0.0037	–0.0050	0.0012	0.0044	–0.0056	0.0002	0.0041
	(0.0064)	(0.0044)	(0.0029)	(0.0065)	(0.0046)	(0.0030)	(0.0066)	(0.0044)	(0.0029)
GDP	0.1017**	0.1261**	0.0940**	0.1295***	0.1423**	0.0935**	0.1048**	0.1332**	0.0945**
	(0.0474)	(0.0622)	(0.0442)	(0.0440)	(0.0643)	(0.0420)	(0.0483)	(0.0673)	(0.0428)
LDR	–0.0199**	–0.0188**	–0.0346***	–0.0244***	–0.0183**	–0.0395***	–0.0222***	–0.0157*	–0.0371***
	(0.0078)	(0.0087)	(0.0074)	(0.0071)	(0.0088)	(0.0070)	(0.0078)	(0.0089)	(0.0072)
Observations	298	298	298	298	298	298	298	298	298
Banks	31	31	31	31	31	31	31	31	31
Instruments	30	30	30	30	30	30	30	30	30
AR(1) test	0.000	0.000	0.000	0.000	0.000	0.000	0.000	0.000	0.000
AR(2) test	0.127	0.248	0.189	0.127	0.367	0.137	0.122	0.391	0.141
Hansen test	0.359	0.449	0.372	0.445	0.469	0.399	0.395	0.463	0.390

Notes: The table presents the results of the GMM in dynamic models. The proxies for business models are NII (column 1–3), HHI (column 4–6), and LLI (column 7–9). The uncertainty measure employed in each regression is shown at the top of each column (UNCA, UNCF, and UNCP). Robust standard errors are in parentheses.

*, **, and *** denote statistical significance at the 10%, 5%, and 1% levels, respectively.

**Fig 2:**
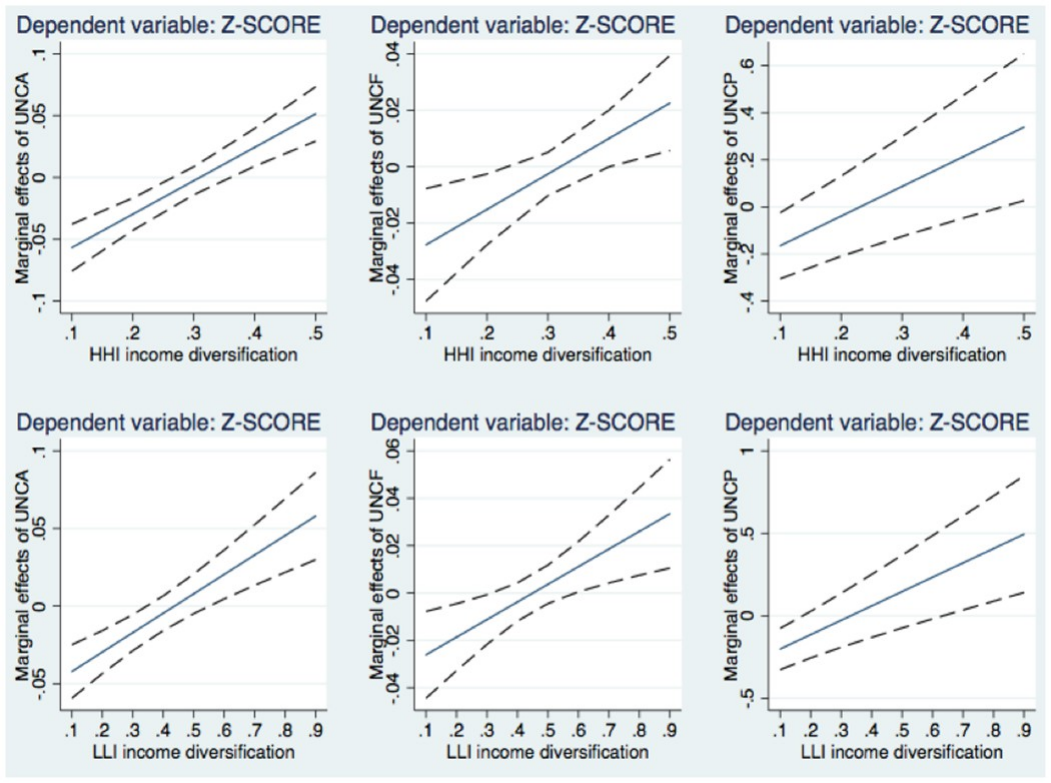
The marginal effects of banking uncertainty on default risk in relation to business models. *Notes*: *The marginal effects according to the HHI measure are calculated with the estimates reported in columns 4–6 of* Table 8, *and those according to the LLI measure are based on the results presented in columns 7–9 of* Table 8.

Fig 2 illustrates the marginal impacts of uncertainty on banks’ default risk, conditional on the level of income diversification based on *HHI* and *LLI*. Similar paths are displayed for the graphs using these two income diversification measures. We observe that the negative effect of uncertainty in banking on *Z-SCORE* is the largest in banks that operate with the lowest level of income diversification. For these banks, a higher level of income diversification can shield their riskiness against an increase in uncertainty. An illustration with the NII income variable also suggests the same result, but we do not illustrate it for brevity. It is available upon request.

In sharp contrast, for banks with a sufficiently high degree of income diversification, the marginal impacts turn positive, i.e., when both the top and bottom bounds of the 95% confidence interval are above zero. At some certain points or higher, an increase in banking uncertainty causes a decrease in bank risk. The graphs indicate the points at 0.365–0.465 for *HHI* and 0.545–0.635 for *LLI* (varying based on the uncertainty measures employed). Accordingly, it is evidenced that those banks with a high level of revenue diversification (about a quarter of the sample) reduce bank risk after uncertainty shocks. A possible explanation is that more diversified business models may reduce banks’ incentives to search for yield since these banks can increase non-interest income to compensate for lost revenues mainly in lending segments. However, when banks earn much enough non-interest income, banks can even adopt the “wait and see” strategy, thus mitigating their risk-taking incentives.

Overall, we document that bank risk tends to increase during higher uncertainty, and business models relying more on non-interest income could mitigate this detrimental impact, which is consistent with Hypothesis 2B; nevertheless, this is not true for banks with the most diversified revenue portfolios. At those banks, the impact of uncertainty in banking on bank risk is insignificant or even turns favorable. We could conclude that diversified business models could serve well as a buffer against bank risk under uncertainty in the banking sector. However, care must be taken when assessing the overall role of diversification strategies since our regressions with standalone income measures indicate that income diversification induces a direct and harmful impact on bank risk. This finding is consistent with Stiroh and Rumble [50], who claim the double-edged nature of the shifts toward non-interest income sources.

### 4.3 Robustness checks

We desire to perform additional checks to strengthen our findings obtained previously. First, in an attempt to validate whether our results still hold when considering diversification across all sources of revenues, in addition to diversification between traditional and non-traditional banking segments, we construct a new measure. This measure is also inspired by the Herfindahl-Hirschman Index (denoted as *HHIall*) for each bank, with total income sources decomposed into five categories: net interest income, income from commissions/fees, income from foreign exchange trading, income from investment in securities, and income from other sources of non-interest income. Hence, the *HHIall* income diversification measure is estimated as follows:

$$HHIall=1–\overset{5}{\underset{i=1}{\sum }}\;{\left(\frac{Income_{i}}{Total\;income\;sources}\right)}^{2}$$ (7)

where *Income*_*i*_ is an income source *i* among five categories as above discussed. As a result, banks that operate with only one or very few revenue sources tend to take a lower value of the *HHIall*. In contrast, banks that supply a broader range of financial services from traditional to non-traditional activities could have a higher value of this diversification ratio, highlighting a more diversified business model. According to previous studies, this approach is also a perfect complement to examine diversification across all business segments of the bank, not just distinguishing between lending and non-lending segments [61,62].

Furthermore, since our sample includes one possible structural break induced by the global financial crisis of 2007–2009, which may drastically impact banking uncertainty and bank behaviors, we modify our sample data by removing this crisis period. We re-estimate our model using the alternative income diversification variable with the new subsample and report the results in Table 9. The results still indicate that banks increase their risk during uncertainty and confirm that income diversification across various sources is also effective in mitigating the detrimental impacts of uncertainty on bank risk. Hence, both diversification “between” (non-traditional and traditional activities) and “across” (various income courses) appear to all reduce the negative impact of uncertainty on bank risk, which is not influenced by the financial crisis.

**Table 9:** Robustness checks with the alternative variable in the new subsample.

	Dependent variable: Loan loss reserves			Dependent variable: Non-performing loans			Dependent variable: Z-SCORE		
	(1) UNCA	(2) UNCF	(3) UNCP	(4) UNCA	(5) UNCF	(6) UNCP	(7) UNCA	(8) UNCF	(9) UNCP
Lagged dependent variable	0.5952***	0.5949***	0.5851***	0.5383***	0.5598***	0.4303***	0.5898***	0.4608***	0.5148***
	(0.0251)	(0.0211)	(0.0259)	(0.0233)	(0.0146)	(0.0364)	(0.0326)	(0.0341)	(0.0304)
Uncertainty	0.0109***	0.0106***	0.1307***	0.0351***	0.0425***	0.5246***	–0.0559***	–0.0465***	–0.2636***
	(0.0024)	(0.0010)	(0.0343)	(0.0082)	(0.0034)	(0.0718)	(0.0143)	(0.0131)	(0.0564)
Uncertainty*HHIall	–0.0093*	–0.0105***	–0.3270***	–0.0906***	–0.0703***	–0.7675***	0.1745***	0.1299***	1.0774***
	(0.0049)	(0.0023)	(0.0793)	(0.0197)	(0.0106)	(0.1596)	(0.0324)	(0.0346)	(0.2508)
HHIall	0.2085***	0.2374***	0.2753***	0.4869***	0.4129***	0.6384***	–6.0794***	–3.9978***	–1.7406***
	(0.0581)	(0.0566)	(0.0754)	(0.1594)	(0.1228)	(0.2328)	(0.5491)	(0.8058)	(0.2657)
Controls	Yes	Yes	Yes	Yes	Yes	Yes	Yes	Yes	Yes
Observations	262	262	262	219	219	219	237	237	237
Banks	31	31	31	31	31	31	31	31	31
Instruments	28	28	28	28	28	28	28	28	28
AR(1) test	0.000	0.000	0.000	0.001	0.002	0.001	0.000	0.000	0.000
AR(2) test	0.716	0.846	0.729	0.141	0.196	0.147	0.134	0.586	0.195
Hansen test	0.227	0.248	0.235	0.182	0.196	0.184	0.487	0.367	0.339

Notes: The table presents the results of the GMM in dynamic panel models. The variables of bank risk are loan loss reserves (column 1–3), non-performing loans (column 4–6), and Z-SCORE (column 7–9). The uncertainty measure employed in each regression is shown at the top of each column (UNCA, UNCF, and UNCP). The alternative variable for business models is HHIall, calculated by the HHI income diversification index across all income sources. Robust standard errors are in parentheses.

*, **, and *** denote statistical significance at the 10%, 5%, and 1% levels, respectively.

Second, we modify our original model by dropping the lagged dependent variable and alter our econometric methodology by using the fixed effects model (proposed by the Hausman test), which identifies the heterogeneity between banks in the sample. We utilize the procedure introduced by Hoechle [63] in fixed effects regressions yielding Driscoll-Kraay standard errors to account for heteroscedasticity, autocorrelation, and cross-sectional dependence issues. We only repeat our regressions using the non-interest income share for the sake of brevity, given that other regressions using income diversification measures all yield supportive evidence for our key patterns. Looking into Table 10, though there appear decreases in the statistical significance level of several estimates, we still obtain key results consistent with our earlier conclusions. Hence, our findings are stable and reliable.

**Table 10:** Robustness checks with the alternative methodology.

	Dependent variable: Loan loss reserves			Dependent variable: Non-performing loans			Dependent variable: Z-SCORE		
	(1) UNCA	(2) UNCF	(3) UNCP	(4) UNCA	(5) UNCF	(6) UNCP	(7) UNCA	(8) UNCF	(9) UNCP
Uncertainty	0.0252***	0.0176***	0.0391	0.0569***	0.0341***	0.1536	–0.0451*	–0.0139*	–0.4391***
	(0.0048)	(0.0051)	(0.0446)	(0.0122)	(0.0083)	(0.1293)	(0.0209)	(0.0069)	(0.0776)
Uncertainty*NII	–0.0001*	–0.0000	–0.0029**	–0.0004	–0.0011***	–0.0211**	0.0006**	0.0002	0.0056
	(0.0001)	(0.0001)	(0.0009)	(0.0005)	(0.0003)	(0.0091)	(0.0002)	(0.0001)	(0.0051)
NII	0.0046***	0.0028**	0.0049***	0.0025	0.0290***	0.0298**	0.0059	–0.0118***	–0.0017
	(0.0009)	(0.0012)	(0.0012)	(0.0154)	(0.0084)	(0.0106)	(0.0046)	(0.0037)	(0.0049)
Controls	Yes	Yes	Yes	Yes	Yes	Yes	Yes	Yes	Yes
Observations	325	325	325	295	295	295	316	316	316
Banks	31	31	31	31	31	31	31	31	31
R-squared	0.251	0.215	0.178	0.197	0.222	0.206	0.192	0.151	0.147

Notes: The table presents the results of fixed-effects regressions with corrected Driscoll-Kraay standard errors in static panel models. The variables of bank risk are loan loss reserves (column 1–3), non-performing loans (column 4–6), and Z-SCORE (column 7–9). The uncertainty measure employed in each regression is shown at the top of each column (UNCA, UNCF, and UNCP). Robust standard errors are in parentheses.

*, **, and *** denote statistical significance at the 10%, 5%, and 1% levels, respectively.

## 5. Conclusion

Taking a further step than the extant literature, we estimate the asymmetric effects of uncertainty on bank risk in relation to the features of business models and plot the results with confidence intervals. Accordingly, considering the difference in business models, we detect that the detrimental uncertainty effect on bank risk is not valid in an identical manner across all banks. We observe that the detrimental uncertainty impact decreases with more non-interest revenue, and it is most pronounced with banks relying on pure lending. Interestingly, a deeper investigation of the graphs reveals that bank risks are immune to banking uncertainty in banks with the highest level of income diversification. In some cases, we exhibit that uncertainty may lower the level of default risk when the income diversification goes beyond a sufficiently high level. These findings contribute to the existing literature by displaying that diversified business models are an efficient buffer against bank risk in periods of high uncertainty.

Our paper yields several policy implications. In the context that uncertainty in banking does have a detrimental impact on bank risk, regulators should be aware of this. They need to take into account policies that can reduce uncertainty and stabilize the banking system. As an illustration, regulators can enhance communication channels with the banking sector, elucidating policy intentions and banking regulations. This proactive engagement promotes a more transparent decision-making process, fostering a predictable and stable financial environment. Given that the adverse uncertainty impact can be mitigated by increasing revenue diversification, banking authorities should construct targeted guidance for banks’ business models in order to provide cushions for the harmful uncertainty effects. For instance, in periods of high banking market uncertainty, a movement toward non-traditional segments and higher levels of income diversification could be encouraged. In other words, regulators could strategically target specific groups of banks, considering differences in income models. This targeted approach ensures that banks can either mitigate the amplification of risk or at least minimize the adverse effects of uncertainty. From the perspective of research implications, it is essential to distinguish between banks with different business models, using the marginal effects with plots constructed, to draw proper conclusions regarding how uncertainty drives bank risk. The implication of our findings holds particular relevance for banks in emerging markets, where non-interest income shares tend to be lower than those in developed markets; nevertheless, these nontraditional segments have experienced proliferation in recent years and continue to present opportunities for further development in the future.

This study acknowledges its focus on a singular market, recognizing the inherent constraints imposed by data limitations. As a caveat, it is advisable to extrapolate the implications of our findings to Vietnam and other emerging countries sharing analogous contextual characteristics rather than making broad generalizations across all countries. For subsequent research endeavors, we advocate extending our analyses to encompass other markets and/or cross-nation samples. Prospective outcomes are poised to either corroborate or challenge the identified patterns, thereby contributing to the nuanced comprehension of the contemporary issue.

## Supporting information

S1 Data (XLS)
